# Development of Plasmonic Attapulgite/Co(Ti)O*x* Nanocomposite Using Spent Batteries toward Photothermal Reduction of CO_2_

**DOI:** 10.3390/molecules29122865

**Published:** 2024-06-16

**Authors:** Shixiang Zuo, Shan Qin, Bing Xue, Rong Xu, Huiting Shi, Xiaowang Lu, Chao Yao, Haoguan Gui, Xiazhang Li

**Affiliations:** 1 Jiangsu Key Laboratory of Advanced Catalytic Materials and Technology, Institute of Urban & Rural Mining, Changzhou University, Changzhou 213164, China; 2 R&D Center of Xuyi Attapulgite Applied Technology, Changzhou University, Xuyi 211700, China; 3 School of Material Science and Engineering, Yancheng Institute of Technology, Yancheng 224051, China

**Keywords:** attapulgite, biochar, spent battery, photothermal catalysis, plasmonic, CO_2_ reduction

## Abstract

The rapid development of the battery industry has brought about a large amount of waste battery pollution. How to realize the high-value utilization of waste batteries is an urgent problem to be solved. Herein, cobalt and titanium compounds (LTCO) were firstly recovered from spent lithium-ion batteries (LIBs) using the carbon thermal reduction approach, and plasmonic attapulgite/Co(Ti)O*x* (H-ATP/Co(Ti)O*x*) nanocomposites were prepared by the microwave hydrothermal technique. H-ATP had a large specific surface area and enough active sites to capture CO_2_ molecules. The biochar not only reduced the spinel phase of waste LIBs into metal oxides including Co_3_O_4_ and TiO_2_ but also increased the separation and transmission of the carriers, thereby accelerating the adsorption and reduction of CO_2_. In addition, H-ATP/Co(Ti)O*x* exhibited a localized surface plasmon resonance effect (LSPR) in the visible to near-infrared region and released high-energy hot electrons, enhancing the surface temperature of the catalyst and further improving the catalytic reduction of CO_2_ with a high CO yield of 14.7 μmol·g^−1^·h^−1^. The current work demonstrates the potential for CO_2_ reduction by taking advantage of natural mineral and spent batteries.

## 1. Introduction

The increasing atmospheric concentration of CO_2_ poses severe threats to the ecosystem. The photocatalytic conversion of CO_2_ into valuable chemicals (e.g., CO, CH_4_, and CH_3_OH) represents a viable solution to combat global warming and tackle energy shortages [1]; however, the CO_2_ conversion efficiency and utilization rate of solar energy remain very low in current research [2]. Notably, the photothermal catalysis with localized surface plasmon resonance (LSPR) has attracted great attention due to its merits in CO_2_ reduction [3]. This is because the LSPR-induced hot charge carriers possess higher energies compared to those generated by conventional photoexcitation [4]. Hence, novel catalysts with optimized microstructures are essential for the development of efficient photothermal catalytic systems. Generally, the catalyst should effectively capture and activate CO_2_ generating electrons and holes with high redox capabilities. Subsequently, various chemicals are formed on the catalyst surface based on their corresponding reduction potentials [5].

Co_3_O_4_ has been regarded as a promising candidate for CO_2_ photothermal conversion, but its intrinsic catalytic activity is unsatisfactory [6]. Co_3_O_4_ nanoparticles with oxygen-rich vacancies exhibit plasmonic effect [7], resulting in the possibility of a highly active and stable Co_3_O_4_ catalyst which can synchronously capture CO_2_ molecules and activate C-O nonpolar bonds in the photothermal catalysis [8]. However, the photothermal abilities of Co_3_O_4_ nanocrystals are restricted due to the rapid recombination of photo-generated hot electron-hole pairs, which can be addressed by the construction of heterostructure [9,10]. As an important resource, the reserves of cobalt are very limited, but on the contrary, huge consumption with about 30% of global production is required by the battery industry [11]. Consequently, the efficient utilization of spent lithium-ion batteries (LIBs) is becoming more important to recycle these solid wastes, in particular as photocatalytic materials [12,13]. As a natural clay mineral rich in magnesium aluminum elements, attapulgite (ATP) is commonly utilized as a solid adsorbent and catalyst support due to its abundant pore structures, rich active sites, and stable chemical properties [14,15]. Furthermore, ATP contains charged oxygen-containing active groups, thus enhancing the interaction between the ATP and CO_2_. ATP has been extensively studied as a catalyst support for CO_2_ conversion in recent years due to its unique advantages [16,17].

In this study, the anode material, primarily composed of LiCoO_2_ and Li_4_Ti_5_O_12_ from LIBs, was utilized to fabricate a novel S-type Co(Ti)O*x* plasmonic heterojunction on the surface of acidified ATP (H-ATP). The resulting H-ATP/Co(Ti)O*x* nanocomposite exhibits LSPR effects in both the visible and infrared regions, effectively activating CO_2_ molecules, which facilitates the capture and conversion of CO_2_.

## 2. Experimental

### 2.1. Carbon Thermal Reduction of LIB Anodes

The LiCoO_2_ and Li_4_Ti_5_O_12_ mixed powder (LTCO, 98 wt%, 160 mesh) of discarded LIB anodes was determined by an Avio 550 Max inductively coupled plasma emission spectrometer (PerkinElmer Corporation, Waltham, MA, USA). The elemental contents are presented in Table 1. Fresh onion powder is subjected to freeze-drying, followed by grinding and sieving for further use.

Subsequently, LTCO and onion powder were uniformly blended with different mass ratios ranging from 1:0.1 to 1:0.9, then the mixed samples were dried at 100 °C for 2 h, and finally the dried samples were heated up to 600 °C at a ramp rate of 10 °C·min^−1^ under nitrogen atmosphere. The temperature was maintained for 20 min, yielding solid samples subjected to thermal treatment. The thermally treated solid samples were dispersed in deionized water and stirred at 80 °C for 6 h, followed by filtration to collect the filter residue. The products were dried at 80 °C overnight and marked as Co(Ti)O.

### 2.2. Preparation of H-ATP/Co(Ti)Ox

ATP was dispersed into 3.0 mol/L of HCl solution with a mass ratio of 1:10, followed by stirring in a water bath for 12 h at 80 °C. Afterwards, the suspension was filtered, washed by deionized water several times, and then dried at 60 °C for 12 h. The acidified ATP (H-ATP) sample was obtained. Subsequently, 0.10 g of Co(Ti)O was added into the H-ATP suspension with different solid contents, and the mixed suspension was poured into a PTFE liner equipped with vigorous ultrasonication, and then the obtained dispersion was transferred into a Teflon-sealed autoclave and maintained at 180 °C for 90 min. The products were separated by centrifugation, washed with deionized water, and dried at 60 °C for 12 h. Finally, the powder was calcined in a muffle furnace at 450 °C for 2 h, and the plasmonic H-ATP/Co(Ti)O nanocomposite was fabricated, denoted as H-ATP/Co(Ti)O-*x*, where *x* represents the various mass ratios of Co(Ti)O to H-ATP (*x* = 10%, 20%, 30%, 40% and 50%). X-ray fluorescence (XRF) analysis was performed on H-ATP/Co(Ti)O-*x* samples to determine their composition and calculate elemental content, as presented in Table 2.

### 2.3. Photocatalytic Reduction of CO_2_

The photothermal catalytic activity of samples was evaluated in a 100 mL photochemical autoclave (Yan Zheng Instrument, Shanghai, China). Initially, 0.1 g of catalyst and 100 mL of distilled water were introduced into the reactor. Subsequently, CO_2_ gas with a purity of 99.99% was continuously injected into the reactor to maintain a pressure of 1.01 bar for 2 min, and then the reactor gas was removed until the pressure was consistent with the atmosphere. The above procedures were repeated three times to guarantee the presence of only pure CO_2_ within the reactor. At room temperature, irradiation (300 W Xe lamp) was directed through a transparent window (diameter: 30~40 mm) at the top of the visible reaction chamber. Throughout the reaction, a syringe was employed to extract the mixed products from the reactor. The reaction products were further measured in a gas chromatograph (Ar as carrier gas) at predetermined intervals (model GC-7860 Plus) with a thermal conductivity detector (TCD).

### 2.4. In Situ DRIFTS Analysis

In situ diffuse reflectance infrared Fourier transform spectroscopy (DRIFTS) analysis of the sample was conducted on a Thermofisher IS-20 instrument (Thermofisher Scietific Co., Ltd., Waltham, MA, USA) under a moist CO_2_ atmosphere and room temperature. At first, 40 mg of catalyst diluted with KBr was introduced into the reaction cell. Pure nitrogen gas was then purged into the reaction cell at a flow rate of 25 mL·min^−1^ for 60 min before measurement. To remove adsorbed contaminants, the sample was preheated under 150 °C in vacuum and subsequently cooled to room temperature. Then, the high-purity carbon dioxide gas was continuously passed into the cells through a water foam apparatus. In the microcell, a CO_2_ + H_2_O gas mixture reached equilibrium of adsorption and desorption, and the experimental data were obtained every 30 min until a steady state was reached. After it reached equilibrium, the catalyst was irradiated with a 300 W Xe lamp, and the infrared spectra were taken every 5 min.

## 3. Results and Discussion

### 3.1. X-ray Diffraction (XRD) Analysis

**Figure 1:**
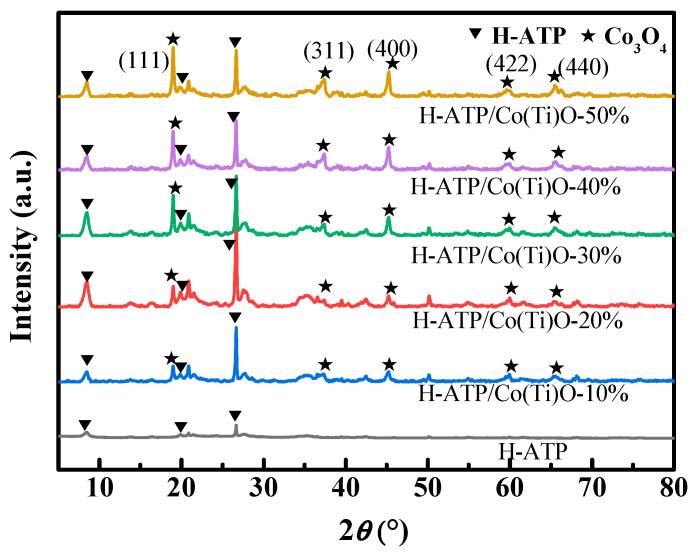
XRD patterns of H-ATP and H-ATP/Co(Ti)O-*x*.

The crystalline structures of the samples are investigated in Figure 1. The peaks of H-ATP at 2θ = 8.3°, 19.7°, and 26.6° correspond to the diffractions of pristine ATP, indicating that acid modification does not destroy its crystal structure. As for H-ATP/Co(Ti)O-*x*, the emerging peaks at 2θ = 18.8°, 36.9°, 45.0°, 59.5°, and 65.3° are attributed to Co_3_O_4_ [18]. However, the diffraction peaks of TiO_2_ can hardly be observed due to its low content (less than 2%, see Table 2). Notably, the intensities of the diffraction peaks of Co_3_O_4_ increase gradually with the increasing content when the Co(Ti)O content is less than 30%. However, when the Co(Ti)O content exceeds 30%, the diffraction intensities of Co(Ti)O slightly enhance while the diffraction intensities of H-ATP decrease significantly, indicating that H-ATP/Co(Ti)O-30% may maintain an optimized composite structure due to the interaction between H-ATP and Co(Ti)O*x*.

### 3.2. Transmission Electron Microscopy (TEM) Analysis

**Figure 2:**
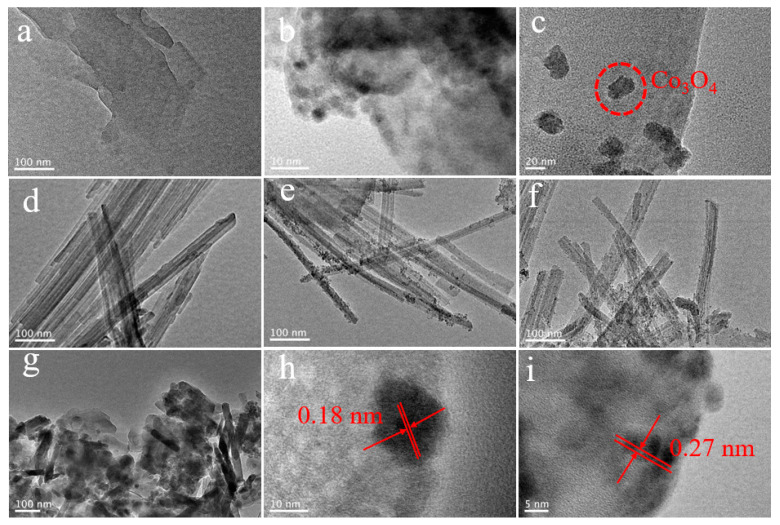
TEM images of (**a**) onion powder, (**b**) LTCO, (**c**) Co(Ti)O, (**d**) H-ATP, (**e**) H-ATP/Co(Ti)O-10%, (**f**) H-ATP/Co(Ti)O-30%, (**g**) H-ATP/Co(Ti)O-50% and HRTEM images of (**h**) Co(Ti)O, (**i**) H-ATP/Co(Ti)O-30%.

The TEM photographs of onion powder, LTCO, Co(Ti)O, H-ATP, and H-ATP/Co(Ti)O-*x* samples are presented in Figure 2. Obviously, the thin slices of onion and clusters of main components in LTCO can be observed, respectively (Figure 2a,b). Co(Ti)O nanoparticles with the average particle size of ~25 nm are uniformly loaded on the surface of the flake-like biochar derived from the onion powder, indicating the effective recovery of Co and Ti compounds from LTCO through the carbon thermal reduction of onion biomass (Figure 2c). The lattice spacing of 0.18 nm can be assigned to the (400) crystal plane of Co_3_O_4_ (Figure 2h). H-ATP exhibits a 1D rod-like morphology after acid modification, suggesting that the lattice structure of ATP is not damaged (Figure 2d), consistent with the XRD results. TEM images of H-ATP/Co(Ti)O-10% and H-ATP/Co(Ti)O-30% (Figure 2e,f) reveal uniformly and highly dispersed Co(Ti)O nanoparticles grown on the surface of H-ATP, and the major particle size of Co(Ti)O greatly is reduced to 1~5 nm. When the Co(Ti)O content exceeds 50%, Co(Ti)O nanoparticles tend to aggregate and cover the surface of H-ATP (Figure 2g), which severely affects the photothermal properties of H-ATP/Co(Ti)O. The HRTEM taken on the edge of H-ATP/Co(Ti)O-30% (Figure 2i), clearly reveals that Co(Ti)O is embedded into the H-ATP nanorod, forming a close contact interface, with lattice spacings of 0.27 nm corresponding to the (220) crystal plane of Co_3_O_4_ [19].

### 3.3. UV-Vis Analysis

**Figure 3:**
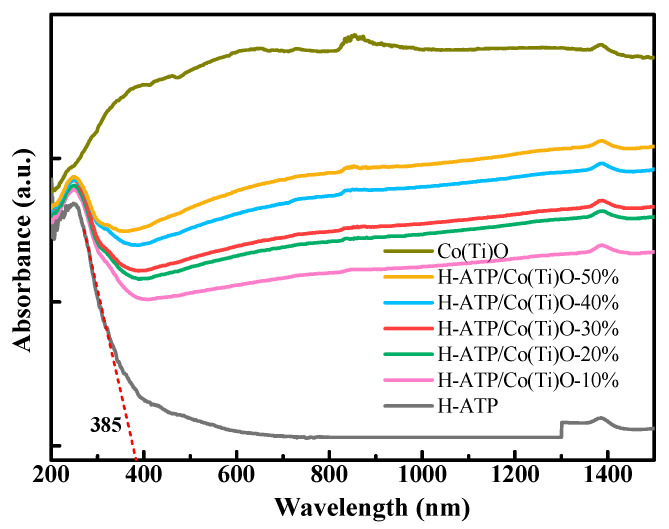
UV-Vis-NIR spectra of ATP, H-ATP, Co(Ti)O, and H-ATP/Co(Ti)O-*x*.

The optical characteristics of the samples are studied using UV-Vis-NIR spectroscopy in Figure 3. The white H-ATP exhibits an absorption edge in the ultraviolet range at 385 nm while the black Co(Ti)O shows full-spectrum absorption. Although the gray H-ATP/Co(Ti)O-*x* falls within the visible light range, especially at 520 nm, the absorption capability extends into the infrared (IR) region, broadening the absorption range of sunlight [20]. The above phenomenon clearly indicates a change in the electronic structure of H-ATP/Co(Ti)O-*x* after the combination of Co(Ti)O with H-ATP, suggesting the possible formation of heterojunctions, further creating an internal electric field. In addition, the heterojunctions can alter the delocalization of photoinduced charge carriers, thereby affecting the separation and migration efficiency of *e*^−^-*h*^+^ pairs [21].

### 3.4. Photoluminescence (PL) and Photoelectrochemical Analysis

**Figure 4:**
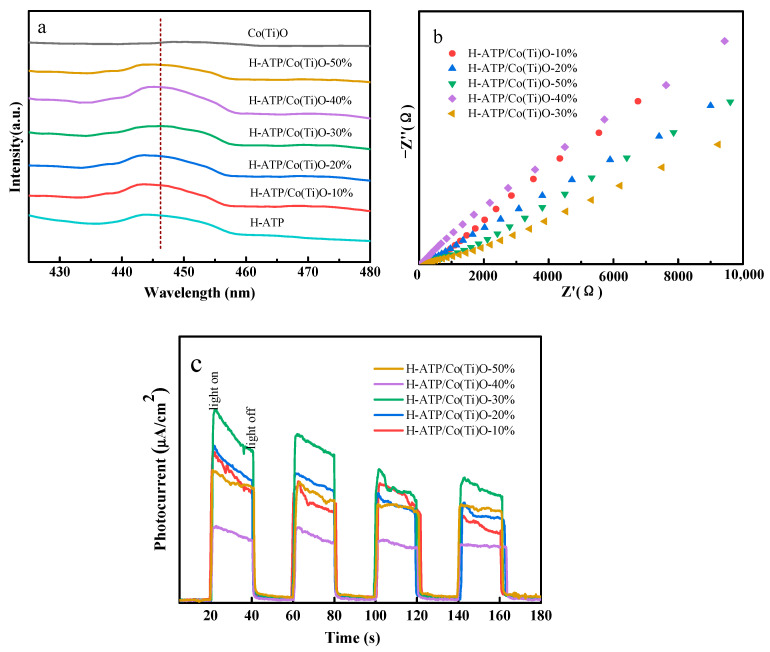
(**a**) PL spectra of H-ATP, Co(Ti)O, and H-ATP/Co(Ti)O-*x*; (**b**) EIS spectra, and (**c**) *I-t* curves of H-ATP/Co(Ti)O-*x*.

The PL and photoelectrochemical spectra are presented in Figure 4. The PL emission peaks are predominantly concentrated around 445 nm (Figure 4a); obviously, H-ATP/Co(Ti)O-30% exhibits the lowest emission peak among all the samples, indicating a higher efficiency in the separation of *e*^−^-*h*^+^ pairs [22]. Compared to the other H-ATP/Co(Ti)O composite, H-ATP/Co(Ti)O-30% exhibits a shorter arc radius (Figure 4b), suggesting lower charge transfer resistance and faster interface electron transfer. Meanwhile, the photocurrent response of ATP/Co(Ti)O-*x* composites is also illustrated (Figure 4c). Under cyclic illumination, H-ATP/Co(Ti)O-30% displays a higher photocurrent density, indicating a superior charge separation-transfer capability and an enormous contact surface between H-ATP and Co(Ti)O [23,24]. The agglomeration of Co(Ti)O particles on the surface of H-ATP nanorods causes a reduction in the efficiency of photoinduced charge separation.

### 3.5. X-ray Photoelectron Spectroscopy (XPS) Analysis

**Figure 5:**
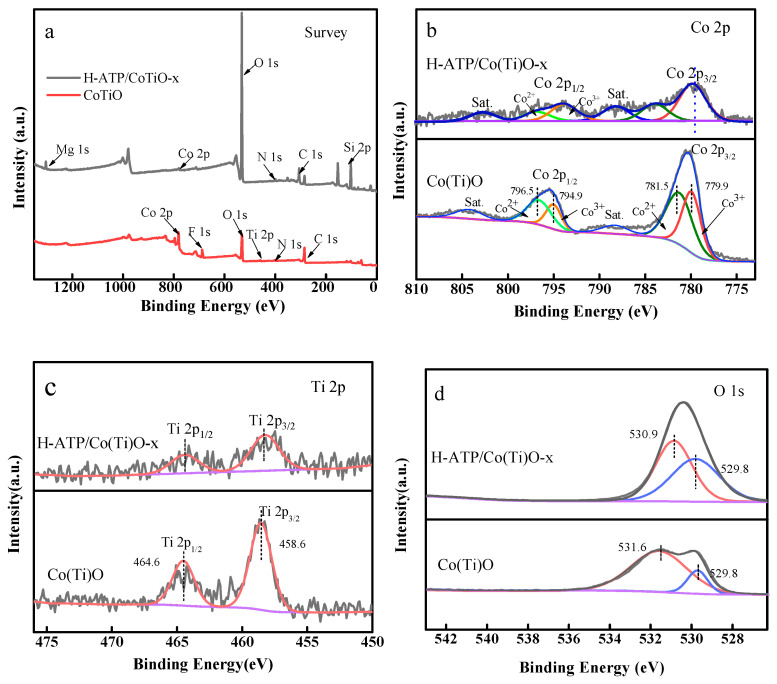
(**a**) XPS survey full scan spectrum of Co(Ti)O and H-ATP/Co(Ti)O-*x*; (**b**–**d**) the high-resolution spectrum of Co 2p, Ti 2p, and O 1s, respectively.

The elemental composition and chemical states of Co(Ti)O and H-ATP/Co(Ti)O-*x* were determined by XPS spectra (Figure 5). The full spectrum of Co(Ti)O reveals the presence of Co, Ti and O elements, as well as F and N elements in onion biochar. For Co(Ti)O, the binding energies (BEs) of 794.9 eV and 796.5 eV correspond to Co 2p_1/2_, and the BEs of 779.9 eV and 781.5 eV belong to Co 2p_3/2_ [25], which are indicative of the simultaneous existence of Co^2+^ and Co^3+^ (Figure 5b). However, the BEs of Co 2p for H-ATP/Co(Ti)O-30% slightly shift towards lower binding energy compared with those of Co(Ti)O, which is due to the fact that the electronegativity of Co (1.88) is weaker than that of Si (1.98)/C(2.55), bringing about a little increase in the electron densities and the screening effect near Co and O. The above phenomenon suggests the formation of Co-O-Si or Co-O-C, which is in good agreement with FT-IR and TEM. The Ti 2p spectrum can be divided into two spin-orbit peaks at BEs of 458.6 eV (Ti 2p_3/2_) and 464.6 eV (Ti 2p_1/2_) (Figure 5c), corresponding to Ti^4+^ in TiO_2_. The peaks around 529.8 eV and 531.6 eV correspond to lattice oxygen and adsorbed oxygen (Figure 5d), respectively [26,27]. Notably, the content of absorbed oxygen in H-ATP/Co(Ti)O-*x* is higher than that of its Co(Ti)O counterpart, suggesting the existence of more oxygen vacancy [28].

### 3.6. CO_2_ Temperature-Programmed Desorption (CO_2_-TPD)

**Figure 6:**
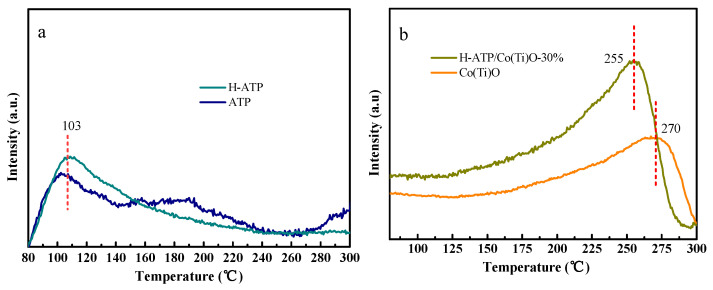
The curves of CO_2_-TPD: (**a**) ATP and H-ATP, (**b**) Co(Ti)O and H-ATP/Co(Ti)O-30%.

The CO_2_-TPD profiles of ATP/H-ATP, Co(Ti)O and H-ATP/Co(Ti)O-30% are shown in Figure 6. Both ATP and H-ATP exhibit a prominent peak around 103 °C, wherein H-ATP displays a larger peak area and stronger CO_2_ desorption intensity, which indicates that acid-modified ATP is conducive to CO_2_ adsorption, attributed to the abundant surface hydroxyl groups and acidic sites, as well as the large specific surface area for CO_2_ adsorption. The desorption temperature of H-ATP/Co(Ti)O-30% (255 °C) is lower than that of Co(Ti)O (270 °C), and moreover, the peak intensity of H-ATP/Co(Ti)O-30% is higher, suggesting a stronger interaction with CO_2_ molecules. Therefore, the H-ATP/Co(Ti)O-*x* facilitates a robust adsorption and activation process for CO_2_ molecules on its surface. Furthermore, the hot electrons generated by the LSPR effect can furnish sufficient charge transfer in the CO_2_ reduction, where the C channel can transport hot electrons, favoring the photothermal CO_2_ reduction [29].

### 3.7. Oxygen Vacancy Analysis

**Figure 7:**
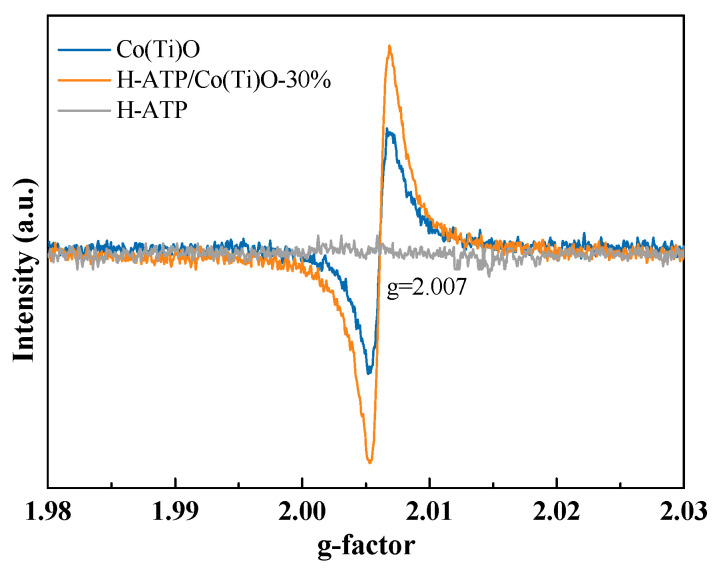
EPR spectra of H-ATP, Co(Ti)O, and H-ATP/Co(Ti)O-30%.

The electron paramagnetic resonance (EPR) spectra for H-ATP, Co(Ti)O, and H-ATP/Co(Ti)O-30% are characterized in Figure 7. No signal is detected for H-ATP, whereas the signals at g = 2.007 for Co(Ti)O and H-ATP/Co(Ti)O-30% are monitored, with H-ATP/Co(Ti)O-30% displaying a stronger signal. This suggests that the number of electrons captured in the oxygen vacancies of H-ATP/Co(Ti)O is more than that in the Co(Ti)O. Oxygen vacancies act as charge carrier acceptors, facilitating interfacial charge transfer and further suppressing the recombination of photoinduced charges [30]. In addition, they have unsaturated points brought by metal coordination, which can favorably contribute to the capture of electrons, enhancing the adsorption and activation of CO_2_, and leading to the conversion of CO_2_.

### 3.8. In Situ DRIFTS Analysis

**Figure 8:**
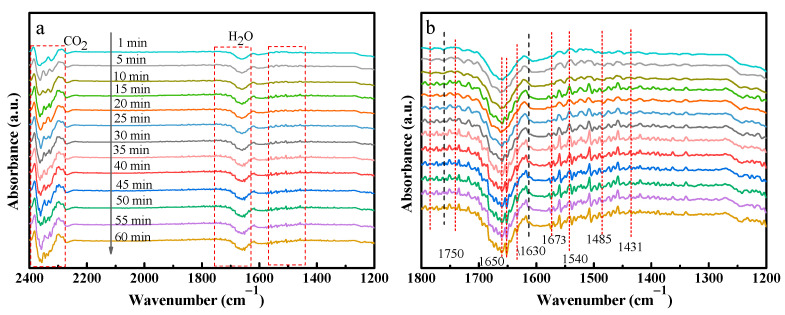
(**a**) In situ DRIFTS of H-ATP/Co(Ti)O for photothermal reduction of CO_2_; (**b**) the partial enlarged section of in situ DRIFTS.

In situ diffuse reflectance infrared Fourier transform spectroscopy (DRIFTS) is employed to explore the reaction pathway of photothermal CO_2_ reduction (Figure 8). As shown in Figure 8a, the peak in the range of 2400–2300 cm^−1^ corresponds to the asymmetric stretching vibration of adsorbed CO_2_, while the absorption band near 1630 cm^−1^ is associated with unbound H_2_O on the surface of H-ATP/Co(Ti)O-30% [31]. Figure 8b is a magnified view of Figure 8a at the wavelength of 1800 cm^−1^~1200 cm^−1^, revealing intermediates detected on the surface of H-ATP/Co(Ti)O-30%. Bicarbonate (HCO_3_^−^) at 1750 cm^−1^ and 1650 cm^−1^, monodentate carbonate (*m*-CO_3_^2−^) at around 1485 cm^−1^, bidentate carbonate (*b*-CO_3_^2−^) at near 1431 cm^−1^, and COOH^−^ at around 1540 cm^−1^ can be observed [32]. Notably, an intermediate ·CO_2_^−^ is detected around 1673 cm^−1^ during the photocatalytic reduction of CO_2_ [33], which is important for the formation of CO product.

### 3.9. Performance of Photothermal Catalytic Reduction of CO_2_

**Figure 9:**
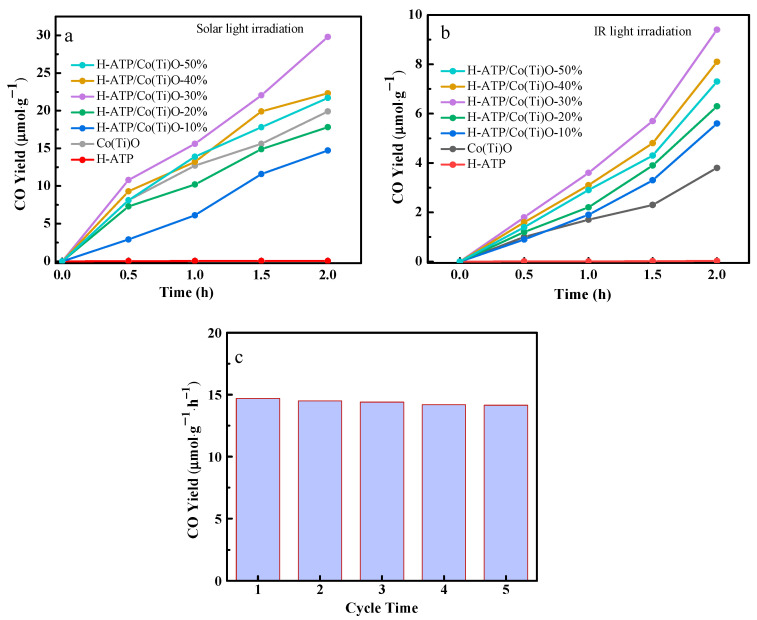
Photothermal reduction of CO_2_ for H-ATP, Co(Ti)O, and H-ATP/Co(Ti)O-*x*. (**a**) Solar light irradiation; (**b**) infrared light irradiation; (**c**) cycling performance under sunlight irradiation.

CO_2_ reduction experiments are implemented under simulated sunlight irradiation. In addition to the CO product, small amounts of CH_4_ and CH_3_OH are also detected, but their yields are very low compared to CO. Under sunlight irradiation, the CO production rate gradually increases with increasing illumination time, and the CO production rates for Co(Ti)O and H-ATP/Co(Ti)O-30% are 9.8 and 14.7 μmol·g^−1^·h^−1^, respectively (Figure 9a). Under infrared irradiation, the CO production rates for Co(Ti)O and H-ATP/Co(Ti)O-30% are much lower at 1.9 and 4.8 μmol·g^−1^·h^−1^, respectively (Figure 9b). H-ATP/Co(Ti)O-30% exhibits the best CO_2_ reduction performance, which is higher than the previous reports [2,10]. On the one hand, Co(Ti)O composed of black Co_3_O_4_, TiO_2,_ and biochar have full-spectrum absorption, are widely used as a photothermal catalytic material [34]. On the other hand, the LSPR effect expands the absorption range from visible to near-infrared light, forming a thermal effect through the release of high-energy hot electrons [3]. After five cycles, H-ATP/Co(Ti)O-30% still maintains a high level of photothermal catalytic performance (Figure 9c).

### 3.10. Mechanism of Photothermal Catalytic Reduction of CO_2_

**Figure 10:**
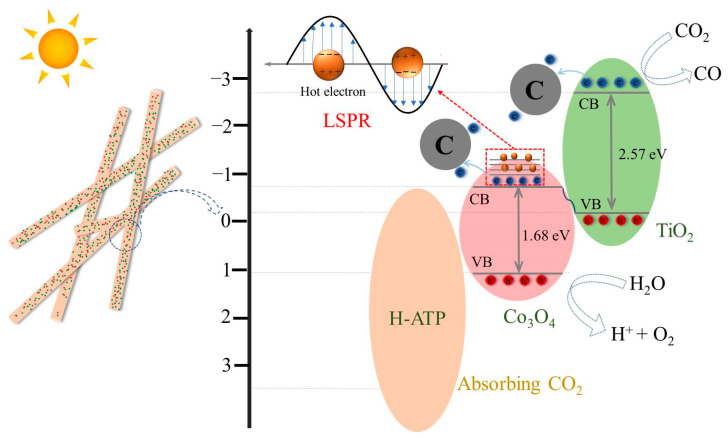
Mechanism of photothermal CO_2_ reduction for H-ATP/Co(Ti)O*x*.

The mechanism of photothermal CO_2_ reduction is illustrated in Figure 10. H-ATP enhances the CO_2_ adsorption capacity because of its large number of active sites. The residual biochar from the carbon thermal reduction of LIB not only reduces the LTCO to metal oxides but also enhances electron transport on the surface of H-ATP, accelerating the adsorption and activation of CO_2_. Under sunlight irradiation, both Co_3_O_4_ and TiO_2_ are simultaneously photo-excited to generate electron-hole (*e*^−^-*h*^+^) pairs. The holes in the VB of TiO_2_ combine with the electrons in the CB of Co_3_O_4_ until their Fermi levels reach equilibrium. Thus, the heterojunction between Co_3_O_4_ and TiO_2_ follows an S-type transfer mechanism. More importantly, the LSPR effect caused by Co_3_O_4_ deposited on the surface of H-ATP enhances the absorption range into the near-infrared region, releasing high-energy hot electrons. The additional heat generated increases the surface temperature of the catalyst, creating a localized environment with high temperature, thereby improving the photothermal activity of the catalyst in the reduction of CO_2_.

## 4. Conclusions

In conclusion, a novel photothermal catalytic reaction system is developed for CO_2_ reduction, which involves the recovery of cobalt and titanium compounds from waste LIB through carbon thermal reduction. The active Co(Ti)O*x* components are uniformly deposited on ATP using a microwave hydrothermal method. The H-ATP/Co(Ti)O*x* composites possess abundant acidic active sites and oxygen vacancies. The introduction of biochar not only reduces the spinel phase of waste LIB to metal oxides but also enhances electron transport, accelerating CO_2_ adsorption and conversion. The LSPR effect caused by Co_3_O_4_ deposited on the surface of H-ATP extends the absorption range into the near-infrared region, thereby enhancing the photothermal catalytic activity for the reduction of CO_2_. Under sunlight irradiation, the main product rate of CO can reach as high as 14.7 μmol·g^−1^·h^−1^ with good stability. This work may provide implications for the recycling of spent batteries for environmental remediation.

## Figures and Tables

**Table 1:** The elemental contents of LTCO.

Element	Co	Li	Ti
Content/wt%	49.69	6.11	2.94

**Table 2:** The XRF results of ATP/Co(Ti)O-*x* samples.

Samples (wt%)	H-ATP/Co(Ti)O-10%	H-ATP/Co(Ti)O-20%	H-ATP/Co(Ti)O-30%	H-ATP/Co(Ti)O-40%	H-ATP/Co(Ti)O-50%
SiO_2_	84.21%	74.91%	65.55%	56.18%	46.82%
Co_3_O_4_	6.34%	13.06%	19.31%	24.79%	32.21%
TiO_2_	0.37%	0.65%	0.98%	1.34%	1.65%

## Data Availability

Data are contained within the article.
